# Examining associations between foundational and complex mathematics skills in people with Down syndrome and typically developing children

**DOI:** 10.1111/bjdp.70034

**Published:** 2026-01-23

**Authors:** Su Morris, Emily K. Farran, Katie A. Gilligan‐Lee

**Affiliations:** ^1^ School of Psychology University of Surrey Guildford UK; ^2^ Social Mobility and Widening Participation Department King's College London London UK; ^3^ Centre for Educational Neuroscience, Birkbeck University of London London UK; ^4^ School of Psychology University College Dublin Dublin Ireland

**Keywords:** Down syndrome, foundational mathematics skills, mathematics profiles, non‐symbolic number skills, symbolic number skills

## Abstract

Acquiring mathematical competence is essential to independent living. In this study, we investigated the mathematics profile in young people with Down syndrome (DS), and the relations between foundational and more complex mathematics skills. The final sample included 33 participants with DS (58% male, 10.2–31.9 years) and a typically developing (TD) group matched on non‐verbal mental age (*N* = 33, 58% male, 4.5–6.5 years). Participants completed mathematics tasks assessing symbolic number, non‐symbolic number, arithmetic, reasoning and geometry. We found that performance was similar on measures of reasoning, non‐symbolic number skills and geometry; however, people with DS showed a relative weakness in symbolic number skills and a relative strength in arithmetic. Associations between foundational skills and more complex mathematics also differed somewhat between groups. These differences may reflect the ongoing role of non‐symbolic skills for mathematics in people with DS and perhaps the use of more complex strategies in typically developing children.

**Statement of Contribution**

**What is already known?**

- Foundational mathematics skills predict performance on more complex mathematics skills in typical development.
- Early mathematics skills in people with Down syndrome may be limited by magnitude comparison and counting skills.
- Little is known about the link between foundational and complex mathematics skills in people with Down syndrome.

**What does this study add?**

- People with Down syndrome have a relative weakness in symbolic number skills.
- There were qualitative differences in the associations between foundational and complex mathematics skills.
- We provide insight into strengths, weaknesses and associations with mathematics in people with Down syndrome.

## INTRODUCTION

Research has shown that foundational mathematics skills are a predictor of later academic success (Aubrey et al., 2006), and of wealth and socio‐economic status in adults (Ritchie & Bates, 2013). In fact, mathematics is an even better predictor of later academic success than literacy (Duncan et al., 2007). Despite the proven importance of mathematics, research rarely extends to understanding mathematics attainment within the context of intellectual disability. In the current study, we address this research gap by focussing on mathematics in individuals with Down syndrome (DS). We determine the profile of strengths and weaknesses across a number of different mathematics tasks in people with DS, relative to typical development and examine relations between foundational number skills and more complex mathematical tasks. Understanding the contributing factors to mathematics success in people with intellectual disability can give insight into how best to support those who struggle, through an understanding of mathematical skill development and the possible role of compensatory mechanisms in the developmental pathways of mathematics skills.

Individuals with DS are born with an additional full or partial copy of chromosome 21, and it is one of the most prevalent neurodevelopmental conditions in England with a prevalence rate of 1 in 377 births in 2020 (NCARDRS, 2022). DS is typically characterised by intellectual difficulties ranging from mild to severe, and deficits in attention, memory and executive function skills (Grieco et al., 2015). There is also evidence that people with DS experience difficulties with both near and distal visual acuity (de Weger et al., 2021), and have a high incidence of nystagmus (Oladiwura et al., 2022) or a squint (von Bartheld et al., 2025) relative to typical development. Mathematics represents an area of difficulty for people with DS compared with typically developing (TD) controls matched for either chronological age or mental age (Brigstocke et al., 2008). It is also an area of relative weakness in childhood and early adolescence in people with DS compared to literacy skills. Typically, mathematics achievement in people with DS tends to be about 2 years behind their literacy level (Buckley, 2007). Most mathematics research with people with DS has focussed on number and arithmetic, and few studies have compared performance across different mathematics subdomains. Our approach of detailing the mathematics profile in DS, across mathematics subdomains, represents the first time such a breadth of mathematics has been examined in people with DS. We also extend current knowledge by examining relations between foundational and more complex mathematics skills. Foundational skills include counting (which involves one‐to‐one correspondence, sequencing number words or ordinality, and knowing the last number is the size of the set or cardinality), recognising small quantities and comparing simple quantities, while more complex skills include calculations, fractions and mathematical word problems.

### Mathematics in typical development

Mathematics is a complex construct with multiple components that requires acquisition and integration of conceptual understanding, factual knowledge and procedural understanding (Gilmore, 2023). There is evidence that early pattern skills (Rittle‐Johnson et al., 2019) and foundational number skills (Sasanguie et al., 2012) contribute to later mathematical success. This is further supported by longitudinal evidence showing that later mathematics attainment is predicted by foundational number skills, even after controlling for domain‐general abilities or demographic factors (Duncan et al., 2007; Geary, 2011), and early growth in mathematics at ages 5–6 years is an even stronger predictor of later mathematics success (Watts et al., 2014). Individual differences in mathematics achievement remain fairly stable through school, such that foundational mathematics skills at school entry predict engagement with mathematics in individuals of college age (Davis‐Kean et al., 2022). This suggests that foundational mathematics skills impact success on more complex mathematics skills in typical development.

Foundational mathematical skills can be categorised as either non‐symbolic or symbolic number skills. Non‐symbolic number skills include the recognition of small quantities without needing to count (known as subitising), focussing on individual items to keep track of them (known as the OTS: object tracking system), and the comparison of (often) larger quantities (known as the ANS: approximate number system) (Leibovich et al., 2017; Porter, 2019). These systems refer to the representation and comparison of numeric magnitude which does not use formal numeric notation; for example, an ANS task may involve the comparison of two groups of dots (Cai et al., 2018; Feigenson et al., 2013). Symbolic number skills refer to the understanding of culturally‐relevant number symbols and words, e.g., the number word ‘three’ and the symbol ‘3’. These symbols do not intuitively relate to a particular magnitude unless that association has been learnt (Kolkman et al., 2013; Scalise & Ramani, 2021).

Associations between symbolic and non‐symbolic number, and competence on more complex mathematics tasks have been extensively examined. There is more consistent evidence supporting the importance of symbolic number compared to non‐symbolic number for later mathematics success (Schneider et al., 2017). A meta‐analysis found stronger associations between ANS and mathematics achievement before the age of 6 years than afterwards (Fazio et al., 2014). This suggests that ANS is an important predictor of mathematical performance for specific age groups only (Inglis et al., 2011), or perhaps only in individuals with lower mathematical ability (Bonny & Lourenco, 2013). For example, it may be the case that the ANS has a particularly important role for individuals who have not yet acquired a robust symbolic number system. In contrast, studies have more consistently revealed positive associations between symbolic comparison tasks and mathematical achievement (Sasanguie et al., 2012). This persists across childhood as evidenced by a meta‐analysis (Schneider et al., 2017), and a longitudinal study (Morsanyi et al., 2020). There is evidence that once improvements in symbolic number skills start to gain momentum at the start of formal schooling, non‐symbolic number skills are no longer a significant predictor of more complex mathematics skills (Lyons et al., 2014). Taken together, it is therefore plausible that non‐symbolic number relates to higher‐level mathematics only until symbolic number skills become more established.

### Mathematics in people with Down syndrome

There is evidence that non‐symbolic number skills are impaired in people with DS (Ansari & Karmiloff‐Smith, 2002). Difficulties for people with DS have been observed relative to mental age comparisons, with matching small quantities using subitizing and comparing quantities where the ratio between dot arrays is small (Karmiloff‐Smith et al., 2012; Sella et al., 2021), although discrimination of larger quantities is in line with mental age comparison groups (Sella et al., 2013). This suggests that foundational mathematics skills in people with DS may be impaired partly due to inherent difficulties in magnitude processing. However, some studies found that individuals with DS (children, adolescents and adults) did not perform significantly differently from mental age matched controls on non‐symbolic magnitude comparison tasks (Abreu‐Mendoza & Arias‐Trejo, 2015; Camos, 2009), that is, the level of deficit was aligned with their overall level of learning difficulties. Further, although adolescents' performance on dot arrays within the subitizing range related to numerical and arithmetic knowledge (Sella et al., 2013), other studies found no association between performance on dot array tasks and other mathematics skills including counting, numeral ordering and basic arithmetic in children and adults with DS (Paterson et al., 2006). In summary, there are mixed findings about whether non‐symbolic number skills are an area of relative weakness in people with DS and how they may impact their more advanced mathematics skills.

Typically, people with DS have difficulty with counting and using the number system. Understanding and remembering the stable order of numbers, and understanding cardinality (i.e., the final number name represents the number of items in that set) are particularly challenging skills to acquire for people with DS (King et al., 2017). In comparison with mental‐age matched TD children, individuals with DS have fewer number words and have shorter counting sequences (Nye et al., 2001). It is also worth noting that some individuals may learn counting sequences by rote, which may not reflect an understanding of the underlying number concepts (Bochner et al., 2002). Difficulties with numeracy may also be due, in part, to impairments in domain general factors, such as language (Brigstocke et al., 2008) and memory (Buckley, 2007). For example, when learning to count, verbal abilities are required to learn number names, and memory is required to remember the number‐name sequence and keep track of number names and objects. Working memory impairments can also lead to difficulties in remembering and using several objects and processes concurrently, which can limit problem‐solving abilities by people with DS (Chapman & Hesketh, 2000).

Impairments in symbolic number skills may have substantial implications for advancement to success in more complex mathematics skills such as learning number facts, arithmetic and word problems (King et al., 2017). For example, there is evidence in the literature of difficulties experienced by people with DS with practical mathematics such as working with money (Bochner et al., 2002) as well as concepts including estimation (Lanfranchi et al., 2015). Despite this, a study with participants with DS aged 8 to 49 years found no significant difference in mathematical reasoning scores compared to a TD comparison group with similar non‐verbal IQ (Simms et al., 2020; Van Herwegen et al., 2020). This suggests that progress in some more complex types of mathematics, such as reasoning, in people with DS is in line with their non‐verbal mental age.

It is possible that people with DS have potential for success across different levels of mathematics which is not in line with a more typical hierarchical progression. By using calculators and other support resources, there is evidence that adolescents with DS can learn more advanced mathematical content such as fractions, percentages and algebra (Martinez & Pellegrini, 2010). There is also some research exploring the use of shapes and relative position in teaching early mathematics to individuals with DS (Gil Clemente & Cogolludo‐Agustín, 2019). The authors report that these early skills could lay the foundation for magnitude comparison, recognising and sorting shapes and number lines, which may otherwise be limited by difficulties associated with counting and magnitude comparison skills. Although further work is needed, the study suggests that children with DS may be able to access more complex concepts using these alternative methods. Together, this suggests that difficulties with foundational mathematical skills can be somewhat compensated for when appropriate support resources and strategies are in place. However, there is currently little research into the attainment of people with DS in more complex mathematics tasks.

Combining evidence across studies, it is difficult to form a clear picture of mathematical strengths and weaknesses in people with DS. There is a need for comprehensive profiles of mathematics skills in people with DS, comparing mathematical sub‐domains from foundational to advanced mathematics skills and including geometry skills which have been omitted from the literature to date.

### Current study

In typical development, there is evidence that foundational mathematical skills relate to success in more complex mathematics (e.g., arithmetic, algebra and problem‐solving). However, far less is known about these relations in people with DS. This could limit the effectiveness of interventions purported to support mathematical development in people with DS. For example, in typical development, the associations between foundational and complex mathematics suggest that there is a hierarchical progression whereby more basic number needs to be mastered before attempting more complex calculations. Due to the known difficulties with basic number skills in people with DS, partly resulting from domain‐general factors such as memory, it is possible that relationships with more complex mathematics may differ. Further, as there has been such a focus on foundational number skills in people with DS, there is little knowledge about performance on other subsets of mathematics, such as geometry. This study will address these gaps through comparing mathematics performance in people with DS and a non‐verbal mental‐age matched group of TD children. There are two research questions:

1. What is the profile of strengths and weaknesses in mathematics (across mathematics sub‐domains) in people with Down syndrome relative to typical development?
2. How do foundational mathematical skills contribute to reasoning, arithmetic and geometry in people with Down syndrome relative to typical development?

## METHOD

### Participants

Participants with DS were recruited via email using the research team's database, and through Down syndrome support groups. Thirty‐six participants with DS were included in the study (58% male, 9.5–31.9 years). However, three of the recruited participants with DS did not complete all the tasks, so were excluded. Specifically, they either did not pass the practice trials for one or more tasks (including the non‐symbolic task and non‐verbal task) or could not complete any question on the numerical operations task. Of the remaining 33 participants (58% male, 10.2–31.9 years), 82% wore glasses and 33% had an additional visual diagnosis (either a squint or nystagmus or both); however, 89% of caregivers (or self‐report for older participants with DS) reported that their vision was average or better‐than‐average. The participants with DS were matched to a TD group (*N* = 33, 58% male, 4.8–6.5 years) using the Ravens Coloured Progressive Matrices test (RCPM; Raven et al., 2003) with a tolerance of ±3 score points. This group will be referred to as the non‐verbal mental age matched TD group (NVMA‐matched). There was no between group difference in RCPM scores, *t*(64) = .946, *p* = .348, *d* = .233; *BF*_*10*_ = 0.369, or verbal scores, measured using the British Picture Vocabulary Scale III task (BPVS‐III; Dunn et al., 2009), *t*(64) = −1.517, *p* = .134, *d* = −.373; *BF*_*10*_ = 0.666. No children in the NVMA‐matched group had a statement of Special Educational Needs and Disabilities (SEND).

See Table 1 for group descriptive statistics.

**TABLE 1:** Descriptive statistics of matched participants by group.

	Variable	Measure	DS group	NVMA‐matched
			*N* = 33	*N* = 33
Age	Age (years)	Mean (SD)	17.4 (5.2)	5.6 (0.5)
		Min–max	10.2–31.9	4.8–6.6
Cognitive scores	RCPM (max 36)	Mean (SD)	17.0 (4.4)	17.9 (3.3)
		Min–max	9–25	11–23
	BPVS (max 168)	Mean (SD)	88.2 (20.7)	81.1 (17.2)
		Min–max	28–126	20–101

Abbreviations: DS, participants with Down syndrome; NVMA‐matched, typically developing group matched using RCPM.

### Procedure

This study was part of a larger project examining spatial profiles and relationships between spatial abilities and mathematics in people with DS (Morris et al., 2024). Procedural details about the study have been outlined previously, and only tasks relevant to the current study are described here. For more information on the broader study procedures see Morris et al. (2024). The study received ethical approval from the University of Surrey Ethics Committee and all participants and parents/caregivers gave their informed consent to take part. Participants completed three groups of tasks: spatial tasks, mathematics tasks, and domain‐general tasks. The DS group completed the RCPM and BPVS online via Zoom to reduce participant fatigue for the face‐to‐face session and enable recruitment of a suitable comparison group (based on the RCPM scores of the group with DS). For the full procedure for online testing, see Morris et al. (2023). In brief, the researcher shared the stimuli via PowerPoint and explained the task instructions. Participants were given the option to either respond by saying the number associated with their choice or by pointing to their choice on‐screen for their caregiver to say the number. Most participants chose to respond by stating the number of their choice. The experimenter recorded these responses and then moved on to the next stimulus. There were no technical difficulties encountered by the participants.

All testing for the NVMA‐matched group took place in a quiet space at their school and face‐to‐face testing for the DS group took place either at the University (*N* = 15) or in their home (*N* = 21). Computer‐based testing used a 19‐inch monitor, and there were practice trials for each task which could be repeated until participants understood the task. The tasks were designed to maintain participants' attention through a game‐based design or object‐based activities. Participants were also able to take breaks as required, which additionally helped to ensure the large test battery was not overwhelming. All instructions and questions were read to the participants by the researcher to ensure there were no effects of reading ability on task understanding. Participants who found it challenging to use a mouse or keyboard responses pointed to the screen for the researcher to complete the response on their behalf. Therefore, no response times were analysed.

### Foundational number skills

#### Non‐symbolic number

**FIGURE 1:**
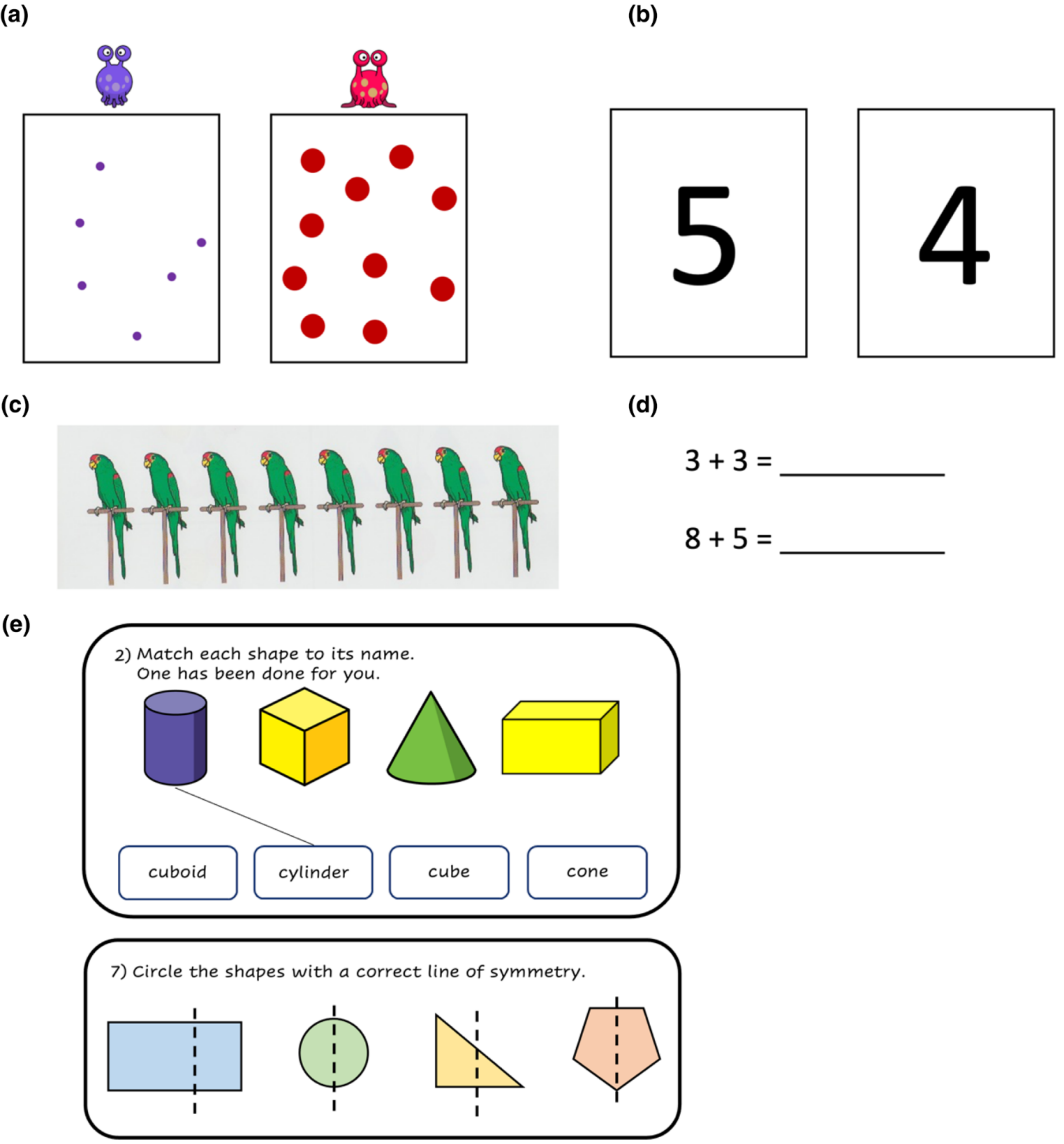
Examples of the mathematics tasks. (a) Non‐symbolic task where participants had to pick which alien had more balls (dots) in their basket (rectangle). (b) Symbolic task where participants had to pick which box (rectangle) had more cake, where the numbers showed the number of cakes in each box. (c) WIAT Mathematical Reasoning; how many parrots? (d) WIAT Numerical Operations example calculations. (e) Geometry.

Participants completed a dot array comparison task, a version of which has been used previously with people with DS (Van Herwegen et al., 2019). The participants were presented with two rectangles, one on the left of the screen and one on the right, that each contained a number of either big or small dots (Figure 1a). The stimuli were described as being two baskets of balls belonging to two aliens, and participants had to choose which alien had more balls in their basket, ignoring the size of the balls. A keyboard press (z or m) was used to make their selection. There were two blocks with 12 trials in each, and correct responses were counterbalanced so that there were an equal number of responses on the left or right of the screen. The ratios between the number of dots were 0.5, 0.6, 0.7 and 0.8, with six trials of each. Half the trials were congruent, that is, the rectangle with larger dots also had a greater number of dots. Each trial was on‐screen for 1500 ms, and there was no time limit. Percentage accuracy was calculated. The Spearman‐Brown split‐half correlation, *r* = .812, indicated good internal reliability.

#### Symbolic number

A symbolic number percentage score was calculated as a mean of two symbolic number tasks: magnitude comparison and counting. The magnitude comparison task, modified from Hawes et al. (2019), was similar in design to the non‐symbolic task, except numerals were used rather than dot arrays (Figure 1b). Participants were presented with two numbers and had to click on the larger number. The stimuli were described as showing boxes with cakes, and the participants had to select which had more cake. This ensured that the language was consistent with the non‐symbolic task, where they were also asked to select which had ‘more’. The numbers ranged from 1 to 9, with mathematical differences between the digits of 1, 2, 3, or 4. There were two blocks of 12 trials, with responses counterbalanced across the task. Percentage accuracy was calculated. The Spearman‐Brown split‐half correlation, *r* = .907, indicated excellent internal reliability.

The counting task was adapted from Ansari et al. (2003) and included numbers up to 20. There were six trials and participants were asked to put a number of dice into a pot (3, 9, 15, 7, 18 and 13). This tested their understanding of number names, one‐to‐one correspondence and cardinality. Percentage accuracy was calculated. The Spearman‐Brown split‐half correlation, *r* = .872, indicated good internal reliability.

### Complex mathematics skills

#### Reasoning

The Mathematical Reasoning subset of the Wechsler Individual Achievement Test‐II (WIAT‐II; Wechsler, 2002) was used to measure participants' ability to reason about age‐appropriate mathematical problems. For each trial, participants saw an image and were then asked a question to which they responded verbally (Figure 1c). Although the task was administered according to the guidelines, which use threshold stopping rules, we started at the first question for all participants, which was an earlier point than the chronological age of participants with DS. Total raw score was calculated. The task has an internal consistency of .92.

#### Arithmetic

The Numerical Operations subset of the WIAT‐II (Wechsler, 2002) was used to measure participants' counting and arithmetic ability. This was a paper‐based task with questions that increased with difficulty as participants progressed (Figure 1d). All participants started at the first question regardless of their age, and the task ended according to the stopping rules in the guidelines. Total raw score was calculated. The task has an internal consistency of 0.91.

#### Geometry

This task comprised questions selected from the White Rose maths assessments (White Rose Education, 2020), and included questions relating to 2D and 3D shapes, coordinates, angles and transformations (Figure 1e). There were 28 questions that covered the UK National Curriculum content for children aged 5–11 years, increasing in difficulty as participants progressed through the task. The task was ended once participants had four successive incorrect or skipped responses, and a raw score was calculated. It was not possible to calculate reliability as participants completed different numbers of questions according to their responses.

### General abilities

Non‐verbal fluid intelligence was measured using RCPM, which has a reported reliability of .90 in TD populations and verbal ability (operationalised as receptive vocabulary scores) was measured using BPVS, which has a reported reliability of 0.91 in TD populations. The tasks were administered according to the guidelines, although the starting set of the BPVS that was administered was lower than participants' chronological age to ensure that an accurate score was calculated for participants with DS.^1^ Raw scores were calculated for both tasks.

### Statistical analyses

A profile of mathematics strengths and weaknesses was created using an analysis of variance (ANOVA), and relations between foundational and more advanced mathematics abilities were evaluated using correlations. The analyses relating to research question 1 were pre‐registered on the Open Science Framework (OSF) as part of the wider study into mathematics and spatial abilities (https://doi.org/10.17605/OSF.IO/JQ3CW). The analyses for research question 2 were not pre‐registered and can therefore be considered as exploratory analyses.

GPower was used to determine sample size. To detect small to medium effects, *N* = 28 was required for the mathematical profile and *N* = 84 was required for the correlations. This means that the correlations were underpowered for detecting small to medium effects. We have reported Bayes factors for non‐significant analyses to show the strength of evidence for the null hypothesis. We have interpreted a BF_10_ between 0.3 and 3 as weak support for either hypothesis. Moderate support for the null hypothesis relative to the experimental hypothesis is assumed for factors of 0.3 to 0.1, and strong support is assumed for factors smaller than 0.1 (van Doorn et al., 2021).

## RESULTS

### Overall task performance

Descriptive statistics for each of the mathematics tasks are presented in Table 2, summarising mean, standard deviation and range for each participant group.

**TABLE 2:** Descriptive statistics by task and group.

Variable	DS group *N* = 33		NVMA‐matched *N* = 33	
	Mean (SD)	Min‐max	Mean (SD)	Min‐max
Non‐symbolic dot array (%)	64.5 (15.6)	41.7–95.8	68.8 (13.0)	41.7–95.8
Magnitude comparison (%)	79.7 (18.0)	41.7–100.0	92.0 (10.0)	62.5–100.0
Counting (%)	78.8 (21.4)	33.3–100.0	87.9 (16.3)	33.3–100.0
Symbolic number composite (%)	79.2 (18.0)	37.5–100.0	90.0 (9.6)	60.4–100.0
Mathematical reasoning (max 72)	28.2 (7.5)	15–46	27.3 (3.1)	21–34
Arithmetic (max 61)	14.8 (5.2)	5–31	11.9 (3.9)	5–18
Geometry (max 34)	6.9 (3.2)	1–17	6.5 (2.5)	2–13

### Profile of mathematical ability

**FIGURE 2:**
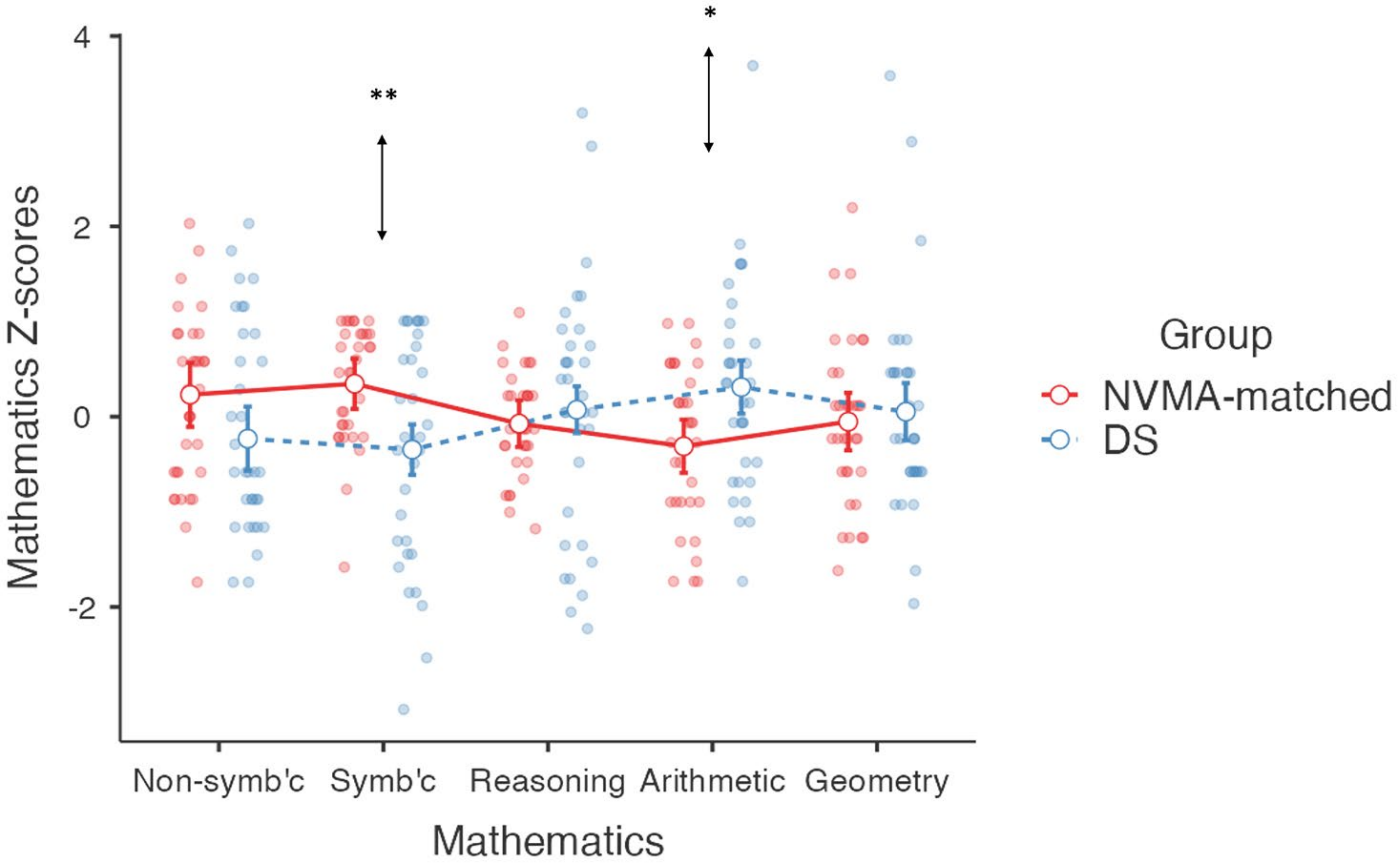
Marginal means and standard errors showing profile of mathematics performance in the DS group compared to the NVMA‐matched group. Non‐symb'c: Non‐symbolic; Symb'c: Symbolic. ***p < .001; **p < .01; *p < .05.

We first compared patterns of mathematics performance between the DS group and the NVMA‐matched TD group. A mixed ANOVA was carried out with *z*‐scored mathematics content (non‐symbolic, symbolic, mathematical reasoning, arithmetic, geometry) as the within‐participant factor and group (DS, NVMA‐matched) as the between‐participant factor. There was no main effect of group, *F*(1,64) = 0.02, *p* = .903, *η* ^2^_*p*_ = .000; BF_10_ = 0.250 and, as all measures were *z*‐scored, there was no main effect of mathematics content. However, there was a significant interaction indicating that the profile of scores across the mathematics tasks varied by group, *F*(4,256) = 8.05, *p* < .001, *η* ^2^_*p*_ = .112. Independent samples *t*‐tests were carried out for each mathematics task to explore the interaction effect. For the symbolic number composite, the NVMA‐matched group achieved significantly higher scores than the DS group, *t*(64) = 3.02, *p* = .004, *d* = .742. In contrast, for the arithmetic task, the DS group achieved significantly higher scores than the NVMA‐matched group, *t*(64) = 2.57, *p* = .013, *d* = .632. All other group differences were non‐significant: Non‐symbolic: *t*(64) = 1.22, *p* = .227, *d* = 0.300, BF_10_ = 0.474; Reasoning: *t*(64) = 0.62, *p* = .537, *d* = 0.153, BF_10_ = 0.297; Geometry: *t*(64) = 0.51, *p* = .612, *d* = 0.125, BF_10_ = 0.282. Although *t*‐test analyses were only powered to detect large effects (*d* = 0.7), the Bayes Factors support the non‐significant findings (Figure 2).

### Relations between foundational mathematics skills and more complex mathematical skills

Pearson correlations were carried out to determine associations between the mathematics measures in each group (Table 3). The sample size is only powered to detect effect sizes of at least 0.47; however, the reported Bayes factors can help in the interpretation of non‐significant associations.

**TABLE 3:** Pearson's correlations between the early and more complex mathematics measures.

	Non‐symbolic	Symbolic	Reasoning	Arithmetic	Geometry
Non‐symbolic		.307 (0.917)	−.164 (0.322)	−.228 (0.471)	−.005 (0.217)
Symbolic	.458**		.326 (1.125)	.394*	.199 (0.390)
Reasoning	.373*	.834***		.649***	.581***
Arithmetic	.451**	.743***	.839***		.376*
Geometry	.142 (0.292)	.613***	.732***	.737***	

*Note*: The Down syndrome group (*N* = 33) is below the diagonal and the NVMA‐matched group (*N* = 33) is above the diagonal. For non‐significant correlations Bayes Factors (BF10) are reported in brackets.

*** *p* < .001.

** *p* < .01.

* *p* < .05.

For individuals with DS, all mathematics measures significantly and positively related to each other, except non‐symbolic number and geometry. In contrast, non‐symbolic number did not relate to any other mathematics measure in NVMA‐matched children, and symbolic number only significantly related to arithmetic in the NVMA‐matched group.

To establish the extent to which these correlations could be explained by general abilities, partial correlations were carried out controlling for BPVS and RCPM scores (Table 4). No Bayes factors were available for partial correlations.

**TABLE 4:** Partial correlations between the early and more complex mathematics measures, controlling for verbal and non‐verbal general abilities.

	Non‐symbolic	Symbolic	Reasoning	Arithmetic	Geometry
Non‐symbolic		.317	−.181	−.221	−.094
Symbolic	.298		.269	.356*	.124
Reasoning	.086	.504**		.552**	.515**
Arithmetic	.259	.457**	.651***		.287
Geometry	.128	.219	.480**	.555**	

*Note*: The Down syndrome group (*N* = 33) is below the diagonal and the NVMA‐matched group (*N* = 33) is above the diagonal.

*** *p* < .001.

** *p* < .01.

* *p* < .05.

For the NVMA‐matched group, the pattern of significant associations remained relatively unchanged compared with the direct correlations. The only change was that the association between geometry and arithmetic was non‐significant when accounting for general abilities. In contrast, for the DS group, the significant direct associations with non‐symbolic number were non‐significant in the partial correlations. Also, the correlation between symbolic number and geometry was non‐significant in the partial correlations.

## DISCUSSION

In this study, we sought to further our understanding of strengths and weaknesses across mathematics sub‐domains in people with DS relative to NVMA‐matched TD children. This is an important gap in our understanding, as there has been little previous research across the breadth of mathematics for people with intellectual disability. Additionally, this study has examined how foundational mathematics skills, as measured by non‐symbolic and symbolic number tasks, relate to more complex mathematics abilities, as measured by reasoning, arithmetic and geometry questions. Together, these findings could feed into future intervention design to determine compensatory routes to learning and to target areas of mathematical weakness in people with DS.

### Research question 1: Mathematics strengths and weaknesses in people with DS

This study provides comprehensive profiles of different sub‐domains of mathematics including number skills, mathematical reasoning, arithmetic and geometry. We found that individuals with DS had a different mathematic profile to an NVMA‐matched group. The DS group performed at a largely similar level to 4‐ to 6‐year‐old NVMA‐matched children on measures of reasoning, geometry and non‐symbolic number skills. The similar performance on the reasoning and non‐symbolic number tasks supports findings in other studies where performance in people with DS was in line with mental‐age matched TD groups (Abreu‐Mendoza & Arias‐Trejo, 2015; Camos, 2009; Simms et al., 2020; Van Herwegen et al., 2020). We also found that our DS group had lower scores on symbolic number skills and higher scores on arithmetic. This relative weakness in symbolic number in people with DS mirrors findings from other studies (Nye et al., 2001) and may be attributable to known verbal weaknesses in people with DS (Brigstocke et al., 2008). Verbal abilities are required to learn number names which are key to success in symbolic number tasks. It is noteworthy that there were no significant differences in receptive vocabulary between the groups in this study; however, it may be that other language skills such as expressive language or comprehension may mediate some of the differences observed. In contrast, the observed relative strengths in arithmetic in the DS group could be experience related. The DS group had completed more years of education (this group was aged 10–31 years) than the NVMA‐matched group, and the arithmetic task primarily measures arithmetic procedural fluency. The DS group will have had greater opportunity to practice procedures for solving written calculations, while the NVMA‐matched group will have only encountered early‐stage arithmetic calculation very recently (in the UK children start school at age 4 to 5 years). However, it is notable that this difference in experience did not confer a similar advantage in the reasoning and geometry tasks. This may be due to the dual challenges of interpreting verbal instruction and applying mathematical understanding in new contexts in the reasoning task, and the mathematical vocabulary required in the geometry task. Additionally, it may be explained by arithmetic being a greater focus in English schools than reasoning and geometry (Department for Education, 2013), which could indicate that repetition and practice are effective in improving mathematics skills in people with DS. Considering the mathematics profile as a whole, there is evidence that mathematics attainment is low in people with DS, relative to chronological age, and specifically, symbolic number is a particular weakness in people with DS. Interventions targeting symbolic number may have extended benefits.

### Research question 2: Associations between foundational and more complex mathematics

Pearson's correlations revealed some distinct patterns of associations between the mathematical measures in each group. In the NVMA‐matched group, non‐symbolic number did not associate with any mathematical measures, and symbolic number only associated with arithmetic. The lack of association between non‐symbolic and symbolic number supports previous research with TD children, which found distinct developmental trajectories of non‐symbolic and symbolic number (Matejko & Ansari, 2016). However, this finding contradicts other research in which a causal link between non‐symbolic and symbolic number in 5‐year‐old children was identified via a training study (Wang et al., 2016). One of the possible mechanisms suggested by Wang and colleagues was that experiencing success on one task resulted in increased self‐efficacy in the mathematical domain, which in turn led to improved outcomes on other mathematical tasks. This may explain our contrasting findings as we gave no feedback on performance. In the current study, symbolic number but not non‐symbolic number associated with arithmetic. This supports suggestions that early mathematical development progresses from discriminating the size of non‐symbolic quantities with increasing precision to relating quantities to symbolic numerals (Siegler & Lortie‐Forgues, 2014), and that the ANS is no longer a significant predictor of more complex mathematics once symbolic number becomes more established (Bonny & Lourenco, 2013; Lyons et al., 2014). Although the literature has found that non‐symbolic number skills in infants and pre‐schoolers relate to mathematical performance in early school age (Libertus et al., 2011), there is a closer association between symbolic number and mathematical attainment at later school ages (Schneider et al., 2017). In fact, symbolic skills (Finke et al., 2020), quantity estimation skills (Libertus et al., 2016), and a counting task (Chu et al., 2015) have been found to fully mediate the association between non‐symbolic and mathematics scores in young children, emphasising the importance of symbolic skills for future mathematics attainment.

The correlations in the NVMA‐matched group remained largely the same even after accounting for general abilities. The non‐significant partial correlation between geometry and arithmetic indicates that either verbal or non‐verbal abilities (or both) explained the significant direct association. It is possible that this is due to the specific language involved in geometry questions, the names of shapes, description of angles (acute, obtuse), features of shapes (sides, vertices) and technical language such as ‘parallel’. As this group were in the early stages of their formal education, it is possible that this pattern will change with development, as they become more familiar with the vocabulary.

Importantly, these patterns were not replicated in the DS group, indicating qualitative differences in the relationships between mathematical tasks between the groups. There were significant associations between all mathematical measures in people with DS except between non‐symbolic number and geometry. The significant associations between non‐symbolic number and the more complex mathematics tasks are a particularly notable difference from the NVMA‐matched group. This may be due to the relatively poor performance in people with DS on symbolic number tasks, which means that the non‐symbolic number skills have not been superseded by symbolic number in the progression towards mastering more complex mathematics. Despite this, symbolic number had a strong association with the more complex mathematics tasks in people with DS, suggesting that those who had developed these foundational skills were able to be more successful on more complex mathematics tasks. This replicates findings from a study with children, adolescents, and adults with DS, where there was a positive association between foundational mathematics performance as measured by a numberline task and more complex mathematics as measured by an arithmetic task (Simms et al., 2020).

Interestingly, we found that non‐symbolic number did not significantly associate with any other task after controlling for general abilities. This shows that verbal and non‐verbal abilities are important factors to account for in research with DS participants that explores associations between ANS and other mathematics tasks. It is also important to note that the association between symbolic number and geometry was non‐significant after accounting for general abilities. As with the NVMA‐matched group, this may be due to the high level of mathematical language involved in the geometry tasks. As verbal abilities are generally an area of relative weakness in people with DS, it could indicate that support with understanding these specialised terms would be beneficial in order to allow people with DS to develop geometry abilities. Additionally, it could indicate an overlap in non‐verbal skills that may contribute to symbolic number skills and geometry attainment, such as spatial skills (Morris et al., 2024).

For both participant groups, these analyses show that counting and numeric magnitude comparisons are important for arithmetic tasks, over and above general abilities. These skills are also important for mathematical reasoning in the DS group. Therefore, effective support for mastering symbolic number skills is important for both TD children and people with DS. As symbolic number is an area of relative weakness in people with DS and relates to both arithmetic and reasoning attainment, this is an area that might show broad benefits for intervention. For example, in a recent systematic review, Van Herwegen et al. (2025) report some success of counting interventions in people with DS but limitations due to the varying methodological rigour of such studies. Alternatively, arithmetic and reasoning may also potentially be enhanced in people with DS if they are provided with resources, such as calculators and other digital tools (Faragher, 2019), to compensate for weak foundational number skills.

### Limitations and future directions

This study investigated the mechanistic associations between foundational mathematics and more complex mathematics. Future studies could include a broader range of mechanisms, such as executive functions (memory, task switching, inhibitory control), to investigate relative contributions. By including both numerical factors and broader domain general abilities, this may provide further qualitative insights on group differences in mathematics performance.

This study was cross‐sectional and participants were individually matched using non‐verbal mental age. Although this meant that the tasks were appropriate for both groups, the groups differed in years of education. A matching method using chronological age may have accounted for years in education, but the scores of the comparison group would have been at ceiling. Future studies could include comparison groups with other genetic conditions, for example, Williams Syndrome. Additionally, training studies would be useful to identify causal associations by comparing effects of training foundational number skills in both TD children and people with DS. This would allow us to evaluate whether any task improvements transfer to progress on more complex mathematics tasks. Furthermore, it would be useful to conduct further work to evaluate performance on mathematics tasks relative to type of school, learning environment, or level of mathematics usage in work. This would allow for an understanding of mathematics cognition in people with DS relative to their mathematical experience and environment.

Finally, future studies could explore different teaching strategies that would best support people with DS develop foundational mathematics skills as well as improve their more complex mathematics attainment. For example, using non‐numeric alternative strategies to develop key foundational concepts in people with DS, such as magnitude comparison, so the progress of mathematics understanding is not limited by difficulties with early number skills.

### Conclusions

This study has established the first profile of mathematics abilities of people with DS relative to a mental age matched control group across a range of both foundational and complex mathematics tasks. People with DS showed a relative weakness in symbolic number skills and a relative strength in arithmetic. There were group differences in direct associations between the foundational and more complex mathematics tasks. Non‐symbolic number was associated with reasoning and arithmetic in people with DS, but did not relate to any other tasks in the TD group. In contrast, symbolic number related to all complex mathematical tasks in the DS group, but only to arithmetic in the TD group. This indicates some qualitative differences between the groups in the relationship between foundational and complex mathematics. After accounting for general ability, the associations between non‐symbolic number and other mathematics tasks, and between symbolic number and geometry were non‐significant in people with DS, which suggests that verbal and non‐verbal abilities were important contributors to these associations. The key remaining group difference was that symbolic number skills related to reasoning and arithmetic in people with DS, but only arithmetic in the TD group. Overall, this study provides valuable insight into strengths, weaknesses, and explanatory factors of mathematics in people with DS, and therefore has important implications for developing future effective support for people with DS.

## AUTHOR CONTRIBUTIONS

**Su Morris:** Methodology; software; formal analysis; investigation; visualization; data curation; writing – review and editing; writing – original draft; project administration. **Emily K. Farran:** Conceptualization; methodology; writing – review and editing; funding acquisition. **Katie A. Gilligan‐Lee:** Conceptualization; methodology; formal analysis; funding acquisition; writing – original draft; writing – review and editing; project administration; supervision; investigation.

## FUNDING INFORMATION

This work was supported by the Baily Thomas Charitable Fund (TRUST/VC/AC/SG/5358–8384).

## CONFLICT OF INTEREST STATEMENT

The authors declare no conflicts of interest.

## Data Availability

The data that support the findings of this study are openly available on the OSF (https://osf.io/dcv8w/files/osfstorage) along with a metadata document explaining each field.

## Footnotes

^1^
The initial BPVS block was selected based on two thirds of the participants' chronological age. If they did not meet the minimum score for the basal block, the researcher would administer a lower starting BPVS block to establish the basal block. The task would then continue according to the guidelines until a basal and ceiling block had been identified and a score could be calculated.
